# SPECC1L-deficient primary mouse embryonic palatal mesenchyme cells show speed and directionality defects

**DOI:** 10.1038/s41598-021-81123-9

**Published:** 2021-01-14

**Authors:** Jeremy P. Goering, Dona G. Isai, Everett G. Hall, Nathan R. Wilson, Edina Kosa, Luke W. Wenger, Zaid Umar, Abdul Yousaf, Andras Czirok, Irfan Saadi

**Affiliations:** 1 Department of Anatomy and Cell Biology, University of Kansas Medical Center, 3901 Rainbow Blvd., Kansas City, KS 66160 USA; 2 Present Address: Clinical Research Training Center, Institute of Clinical and Translational Sciences, Washington University, St. Louis, MO USA; 3 Present Address: Center for Regenerative Medicine, Massachusetts General Hospital, Harvard Medical School, Boston, MA USA

**Keywords:** Mechanisms of disease, Cell migration, Time-lapse imaging, Disease model

## Abstract

Cleft lip and/or palate (CL/P) are common anomalies occurring in 1/800 live-births. Pathogenic *SPECC1L* variants have been identified in patients with CL/P, which signifies a primary role for SPECC1L in craniofacial development. *Specc1l* mutant mouse embryos exhibit delayed palatal shelf elevation accompanied by epithelial defects. We now posit that the process of palate elevation is itself abnormal in *Specc1l* mutants, due to defective remodeling of palatal mesenchyme. To characterize the underlying cellular defect, we studied the movement of primary mouse embryonic palatal mesenchyme (MEPM) cells using live-imaging of wound-repair assays. SPECC1L-deficient MEPM cells exhibited delayed wound-repair, however, reduced cell speed only partially accounted for this delay. Interestingly, mutant MEPM cells were also defective in coordinated cell movement. Therefore, we used open-field 2D cultures of wildtype MEPM cells to show that they indeed formed cell streams at high density, which is an important attribute of collective movement. Furthermore, activation of the PI3K-AKT pathway rescued both cell speed and guidance defects in *Specc1l* mutant MEPM cells. Thus, we show that live-imaging of primary MEPM cells can be used to assess mesenchymal remodeling defects during palatal shelf elevation, and identify a novel role for SPECC1L in collective movement through modulation of PI3K-AKT signaling.

## Introduction

Development of secondary palate involves coordinated growth and movement of palatal shelves in conjunction with surrounding craniofacial structures^1–3^. In mice, the palatal shelves originate as a pair of vertical outgrowths from the maxillary processes at embryonic-day 11.5 (E11.5) and extend downward adjacent to the tongue until E13.5^1^. By E14.5, the palatal shelves elevate to position themselves horizontally above the tongue and extend toward the midline. By E15.5, the shelves have adhered and fused at the midline to form the secondary palate. Defects in palatal shelf outgrowth, elevation, or fusion can lead to cleft palate, one of the most common human birth defects^1,4^. Among these steps, elevation is the most poorly understood. Histological studies of palate elevation have shown that the mechanism differs along the anteroposterior axis^1,5,6^. The anterior palate exhibits a “flipping-up” motion where the distal ends of the shelves rise toward the midline. In contrast, the middle and posterior sections of the palate undergo more extensive remodeling, wherein the medial walls of the shelves extend horizontally as the distal ends of the shelves retract, creating a “bulge”^5–7^. This latter process can be referred to as mesenchymal remodeling^5^. Interestingly, the “bulging” appears to occur first, indicating it may be the driving event during elevation^6^.

Several cellular mechanisms for mesenchymal remodeling during palate elevation have been proposed^1,2,8,9^. Cell proliferation is required, but not sufficient, for elevation to occur^10–12^. Organ culture of palatal shelf explants have shown that palatal mesenchyme also has migratory properties, potentially guided by WNT5A and FGF10 chemotactic gradients^13^. Thus, coordinated movement of the palatal mesenchyme may contribute to palate remodeling, however, collective migration attributes have not been investigated in palate mesenchymal cells.

SPECC1L is a cytoskeletal protein that associates with both filamentous actin and microtubules^14^. Mutations in *SPECC1L* have been identified in multiple patients with craniofacial malformations, including cleft palate^14–17^. Thus far, these mutations have been autosomal dominant nonsynonymous variants that cluster in the second coiled-coil (CCD2) and the C-terminal calponin homology (CHD) domains^15^. Mouse embryos homozygous for a null *Specc1l* gene-trap allele die between E9.5 and E10.5 with open neural-folds and defects in cranial neural crest cell delamination^18^. In cultured U2OS osteosarcoma cells, loss of SPECC1L results in poor cell migration in wound-repair assays^14^, increased filamentous actin staining^14,18^, and at high cell-density, cell-shape change and abnormal staining of adherens junction markers β-catenin and E-cadherin^18^. We have recently reported generation of a new gene-trap allele (*Specc1l*^*cGT*^), and a *Specc1l*-truncation allele lacking 510 C-terminal amino acids (*Specc1l*^*∆C510*^) that removes the CHD. Homozygous mutants for *Specc1l*^*cGT*^ are embryonic lethal, while those for *Specc1l*^*ΔC510*^ are perinatal lethal^19^. Crossing these alleles resulted in *Specc1l*^*cGT/∆C510*^ compound heterozygous mutant embryos that were also perinatal-lethal and exhibited a delay in palate elevation as well as oral epithelial defects^19^. In particular, we reported the presence of transient oral epithelial adhesions between palatal shelf and tongue or buccal surfaces, and ectopic expression of adhesion molecules at the apical surface of the palate periderm layer^19^. As SPECC1L is broadly expressed in both palate epithelium and mesenchyme, we hypothesized that it may also play a role during mesenchymal remodeling.

In this study we used quantitative analyses of motility to show that primary mouse embryonic palatal mesenchyme (MEPM) cells exhibit stream formation, an attribute of collective migration, and showed that this behavior is impaired in *Specc1l*-mutant MEPM cells. We also performed wound-repair experiments using primary MEPM cells from *Specc1l*^*cGT/∆C510*^ mutant embryos to show defects in both cell speed and directionality. Importantly, we show that pharmacological activation of the PI3K-AKT pathway, which is reduced in *Specc1l* mutants, is sufficient to rescue speed and directionality defects in these cells. Together, these data show a novel role for SPECC1L in collective cell movement through regulation of PI3K-AKT signaling pathway, as well as establish MEPM cells as a proxy model to study mesenchymal remodeling defects during palate elevation.

## Materials and methods

### MEPM isolation and cell culture

*Specc1l*^*ΔC510/*+^ × *Specc1l*^*cGT/*+^ mouse mating-pairs were placed together overnight, and the gestational stage was defined as E0.5 at noon on the day plug was identified. Females were euthanized at E13.5, and embryos were harvested in 1× PBS. Palatal shelves were dissected away from the oral cavity under aseptic conditions, placed in a 1.5 mL tube with 0.5 mL of 0.25% Trypsin (ThermoFisher, 25200056), and incubated at 37 °C for 10 min. Occasional pipetting was performed to accelerate mechanical dissociation. The trypsinized MEPM cells were mixed with 5 mL of high-glucose DMEM (HyClone, SH30243.01) containing 10% FBS (Corning, 35-010-CV) in a conical tube. The tube was centrifuged at 200rcf for 5 min to pellet the cells. MEPM cells were resuspended in fresh DMEM containing 10% FBS, plated on tissue-culture treated plastic dishes, and incubated at 37 °C. Following expansion, cells were cryopreserved, and only passaged up to 3 times for use in experiments. All experiments involving animals were carried out with a protocol approved by the KUMC Institutional Animal Care and Use Committee, in accordance with their guidelines and regulations.

### U2OS cell culture

Control and *SPECC1L*-kd U2OS cells were generated previously from U2OS osteosarcoma cells (ATCC HTB-96) as reported^14^. U2OS cells were cultured in DMEM (HyClone, SH30243.01) containing 10% FBS (Corning, 35-010-CV) as shown previously^18^.

### PI3K-AKT pathway activator treatment

For PI3K-AKT pathway small-molecule activation experiments, 100 µg/mL 740Y-P (ApexBio, B5246) was added 24 h prior to imaging. The activator containing medium was refreshed at the onset of imaging, and it remained throughout the duration of the experiment (48 h). An equal proportion of water was added as vehicle to control wells.

### Live-cell imaging

Imaging was performed using the EVOS FL Auto Imaging System (ThermoFisher, AMAFD1000) with the EVOS Onstage Incubator (ThermoFisher, AMC1000). Cell cultures were kept in a humidified, 5% CO_2_ environment at 37 °C over a period of 48–72 h. Phase contrast images were collected at × 4 or × 10 magnification, every 10 or 20 min.

### Image processing

To detect cell-occupied area, a global threshold was applied to the local standard deviation of image brightness following the procedures outlined in Neufeld, et al.^20^. The code is available at http://github.com/aczirok/cellconfluency. Cell motility was extracted using our particle image velocimetry (PIV) algorithm, with an initial window size of 50 µm^21,22^, resulting in a velocity field *v(x,t)* for each frame *t* and image location *x*. The average speed of cell motility was extracted from *v(x,t)* as a spatial average over the cell-occupied area.

### Wound-repair assay

For wound-repair assays, MEPM and U2OS cells were seeded at superconfluent densities of 1400/mm^2^ and 2300 cells/mm^2^ (respectively) into silicone inserts (Ibidi, 81176) in 35 mm diameter tissue culture dishes. These silicone inserts provide 500 µm wide cell-free gaps. Live-imaging started at the removal of the silicone insert, 48 h after cell attachment (56 h after seeding). For each wound, 4–5 non-overlapping fields were recorded every 10 min at × 10 objective magnification. Image sequences were evaluated by compiling confluency data *A(t)*—the size of cell-occupied area normalized to the field of view, as a function of time. The speed of wound-closure was established as *V* =  (*w*/2) *(dA/dt)*, where *w* denotes the width of the field.

### Spontaneous collective motility assay

For the analysis of general stream formation and collective motility of MEPM cells, we first estimated the effective density of cells migrating into the wound. This density of cells migrating into the wound along the cell-front after 14-h of migration was approximately 600 cells/mm^2^. Therefore, we started by seeding cells at a lower density of 300 cells/mm^2^, allowed them to grow for 20 h, and then live-imaged these cells for 30 additional hours. This time-frame allowed us to capture the desired “high” density of 600 cells/mm^2^ that we found in the migratory cell-front of wound assays. We then seeded cells at densities of 100 cells/mm^2^ (“medium”) and 30 cells/mm^2^ (“low” or individual cells) for comparison. MEPM cells were seeded at low (30/mm^2^), medium (100/mm^2^), and high (300/mm^2^) densities into 3D-printed 6 mm diameter rings on 35 mm tissue culture dishes^23^. Following overnight growth, cultures were live-imaged every 10 min with a 4× phase contrast objective for and additional 30 h.

Local spatial correlations of cell movements were characterized by the average flow field that surrounds moving-cells as described previously^24,25^. Briefly, a reference system was aligned to each vector *v*(*x,t*) and their “neighbors” registered in the appropriate bin (front, rear, etc.). The average velocity vector (*U*) in each bin is indicative of spatial correlation: the average of random vectors is close to zero, while it is non-zero when velocities share a common component. The calculated *U(x)* flow field was fitted with an exponential function *U(x)* = a*exp(− *x/* × 0*)* + *U*0, where × 0 is the correlation length: the characteristic distance at which local velocity–velocity correlations disappear.

### Nuclear orientation assay

To quantitatively characterize local orientational ordering of mesenchyme nuclei, we performed the following image analysis sequence: (1) DAPI-labeled frozen sections were imaged with × 20 objective magnification. (2) After loading the images into ImageJ^26^, the palatal shelf area was delineated, then nuclei were segmented by brightness-based global thresholding. (3) Touching nuclei in the binary segmented image were resolved by a watershed transformation. (4) Segmented clusters larger than 10 pixels were identified and fitted with an ellipse. (5) Nuclei with an unambiguous orientation (the ratio of the minor and major ellipse axes being less then 0.8) were assigned into cells of a spatial grid with a cell size of 50 um. (6) In each cell, we calculated the scalar 2D nematic order parameter^27^ as ${S}^{2}= {\langle \mathrm{cos}2\theta \rangle }^{2} + {\langle \mathrm{sin}2\theta \rangle }^{2}$, where theta ($\theta$) is the angle between the long axis of the ellipse and a reference direction, and the averages $\langle ..\rangle$ are calculated for each nucleus within the grid cell. The order parameter S = 1 indicates a configuration in which nuclei are completely parallel, while S = 0 indicates a completely random set. Intermediate numbers 0 < S < 1 indicate various degrees of partial ordering. Using the same data, we also calculated the direction of the local prevailing order. (7) We used the same measures to compare regions within the palatal shelves by pooling the segmented nuclei from multiple specimens into three spatial domains (lingual, buccal and hinge).

### Analysis of individual cell-trajectories

Individual cells were tracked manually on consecutive images (github/donnagreta/cm_track) yielding positions *P(i,t)* of cell *i* at time *t*. Motivated by a similar analysis performed by Biggs et al.^28^, trajectories were characterized by the total path-length $T\left(i,t\right)= \sum_{t`=0}^{t}\left|P\left(i,t`+1\right)-P(i,t`)\right|,$ net displacement into the wound *D(i,t)* =*|X(i,t)-X(i,*0*)|*, where *X* is the projection of *P* in the direction perpendicular to the wound. Guidance efficiency was calculated as *D(i,t)/T(i,t)* for each cell *i* and timepoint *t*. Cultures were characterized by the population average of these single cell measures, evaluated at a suitable timepoint *t*.

### Statistical analysis

To establish statistical significance, we calculated the quantitative measure for each independent sample (time-lapse recording, physical section of an embryo). The sets, containing at least 4 independent values, were compared by two-tailed Welch t-tests, which does not assume equal variance or paired data. For the calculations we used the scipy.stats.ttest_ind function of the python programming language.

## Results

### ***Specc1l***^***cGT/ΔC510***^ mutant embryos show abnormal palatogenesis

**Figure 1:**
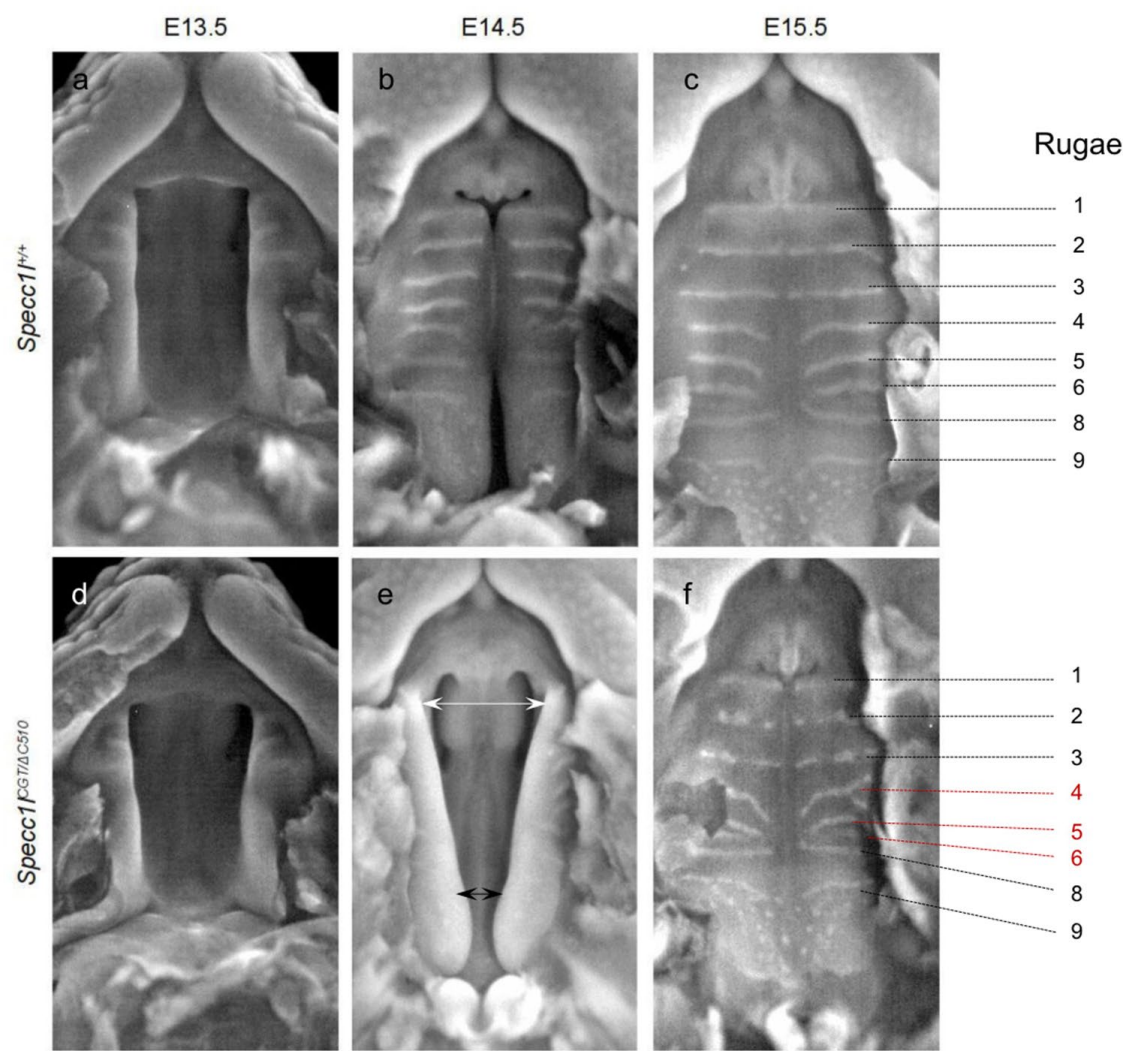
*Specc1l* deficiency results in abnormal palate closure and rugae formation. Compared to wildtype (**a**–**c**), *Specc1l*^*cGT/ΔC510*^ mutant embryos (**d**–**f**) exhibit abnormal palatal shelf elevation ((**b**) vs. (**e**); arrows). By E14.5, normal palate elevation results in apposition of the anterior to middle regions of the palatal shelves (**b**). In contrast, at E14.5, mutant shelves show greater elevation posteriorly ((**e**); black arrows) rather than in anterior and middle regions ((**e**); white arrows). Although by E15.5, the mutant palatal shelves manage to elevate and adhere, they still show defects in rugae formation (**f**). These rugae patterning defects include both discontinuous and missing rugae ((**c**) vs. (**f**)), which persist until birth. Abnormal palatal shelf elevation sequence suggests a role for SPECC1L in mesenchymal remodeling during this process.

**Figure 2:**
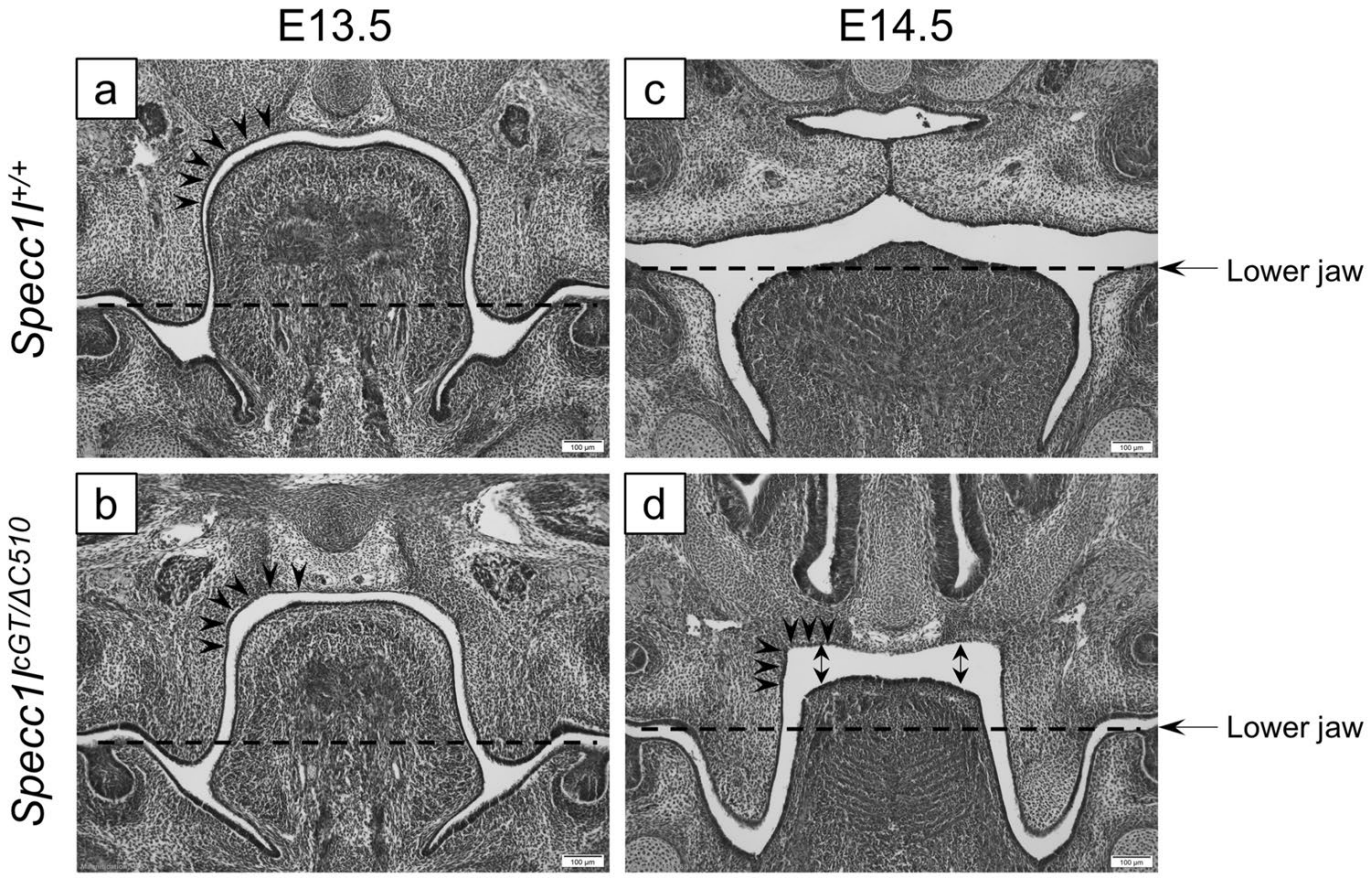
Abnormal *Specc1l*^*cGT/ΔC510*^ mutant palatal shelf morphology at E14.5. (**a**–**d**) Histological sections from wildtype (**a**,**c**) and *Specc1l*^*cGT/ΔC510*^ mutant (**b**,**d**) embryos at E13.5 (**a**,**b**) and E14.5 (**c**,**d**) are shown. At E13.5, wildtype palatal shelf presents with a rounded curve around the tongue ((**a**), arrowheads). This curvature in the mutant is already more angular at E13.5 ((**b**), arrowheads), and becomes much more acute at E14.5 ((**d**), arrowheads) during the delayed shelf elevation. The depression of the tongue from E13.5 to E14.5 in the mutant appears adequate when compared to the level of the lower jaw ((**a**–**d**); dashed line). At E14.5 in the mutant, there is increased space above the tongue ((**d**), double-arrows), but the palatal shelves have not moved into the space.

*Specc1l*^*cGT/ΔC510*^ compound heterozygotes are perinatal lethal and show a delay in palate elevation at E14.5^19^. However, palatal shelves in most mutants recover and fuse by E15.5 (Fig. 1)^19^. Analysis of E14.5 sections at mid-palate region indicated that the tongue in the mutant is usually adequately depressed, but the palatal shelves are acutely angled and do not immediately move inwards to occupy the space above the tongue (Fig. 2)^19^. Importantly, palate elevation in these mutants follows an abnormal sequence where posterior palate advances prior to middle and anterior palates, as in wildtype (WT) embryos (Fig. 1b vs. e). Given the delayed and abnormal palate elevation in *Specc1l*^*cGT/ΔC510*^ mutants, we hypothesized that (a) this delay is due to poor mesenchymal remodeling during elevation, and (b) coordinated movement of palatal mesenchyme cells is required for efficient remodeling. To determine the role of SPECC1L in the mesenchymal remodeling during palate elevation, we decided to perform in vitro motility assays using primary mouse embryonic palatal mesenchyme cells (MEPM) from WT and *Specc1l*^*cGT/ΔC510*^ compound heterozygous embryos.

### *Specc1l*-deficient mouse embryonic palatal mesenchyme (MEPM) cells show migration defects during wound-closure

**Figure 3:**
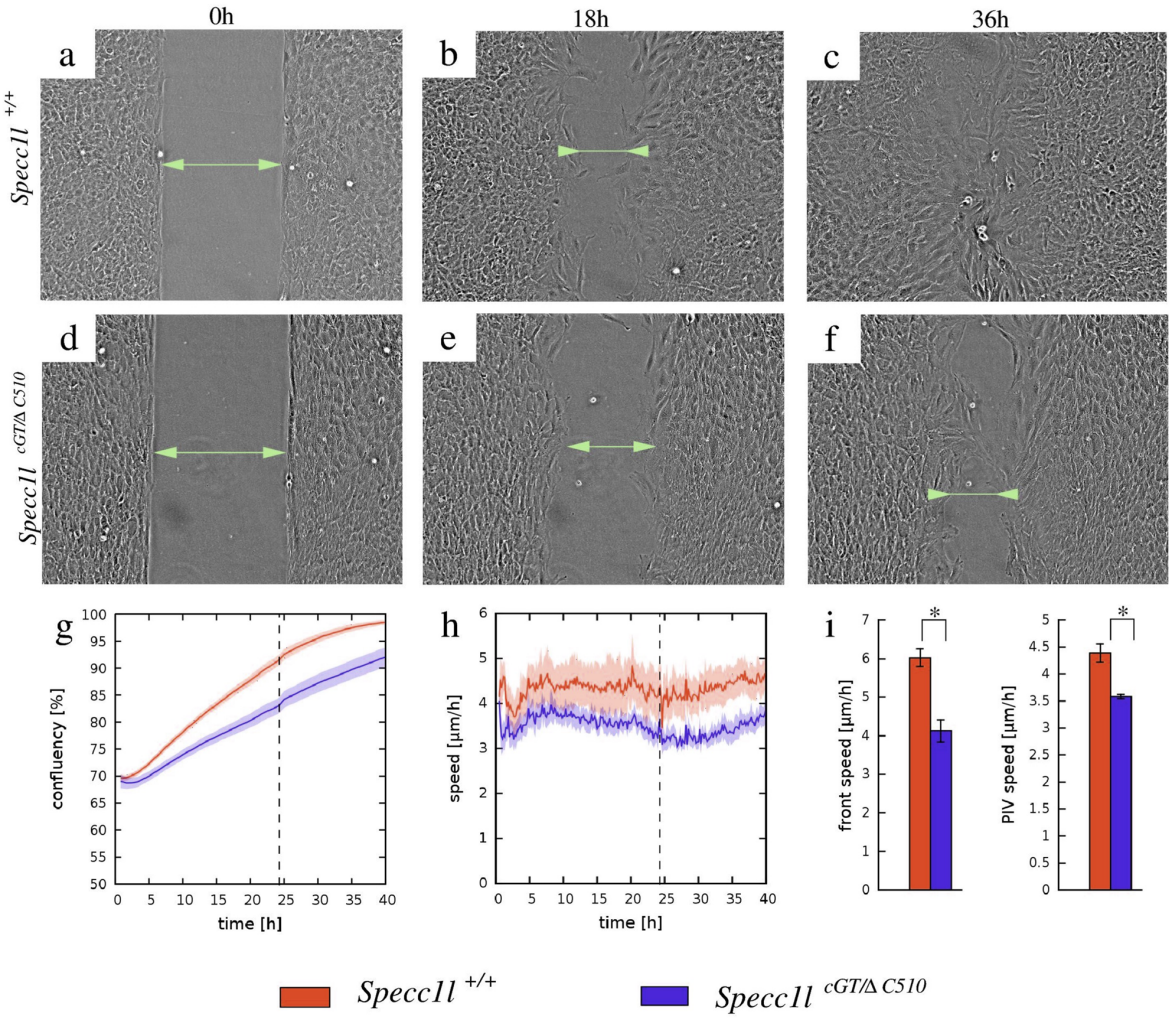
*Specc1l*^*cGT/ΔC510*^ primary MEPM cells exhibit migration defects in wound-repair assays. (**a**–**f**) Phase contrast micrographs of a representative wound-repair assay performed with wild-type (WT) and *Specc1l*-mutant MEPM cells. While WT cells (**a**–**c**) show complete wound-closure by 36 h (h), the mutant cells (**d**–**f**) require more time. The wound area is indicated by two-headed arrows. (**g**) Automated confluence analysis of wound-repair experiments quantified the average percentage of wound-closure over time. Orange and blue colors indicate data obtained from WT and mutant cultures, respectively. By 24 h (dashed vertical line), WT MEPM cells close more than 66% of the wound. Solid lines represent the average of 29 independent microscopic fields, recorded in 5 independent experiments. Shaded areas indicate SEM. (**h**) Average cell motility speed, calculated by PIV analysis of the 29 wound-closure recordings analyzed in (**g**). For each time point velocity magnitudes within the entire cell-covered area were averaged. Despite approaching wound-closure after 24 h (dashed line), cells remained motile in both WT and mutant MEPM cultures. (**i**) Statistical analysis of data presented in (**g**,**h**) showed that the motility of mutant MEPM cells is significantly reduced in comparison to WT MEPM cells. Left: average speed of wound front, calculated within the 1–15 h time interval (p < 6.7 × 10^−6^, Welch’s t-test). Right: average speed of cell movements within the entire recorded time period of 40 h (p < 2.2 × 10^−5^, Welch’s t-test). Error bars represent SEM.

We previously reported that SPECC1L-deficient U2OS osteosarcoma cells showed poor migration in wound-repair assays^14^. To determine if MEPM cells from *Specc1l*^*cGT/ΔC510*^ compound heterozygous embryos showed similar defects, we live-imaged wound-repair assays at superconfluent cell densities (Suppl. Movie 1). As representative images of time-lapse recordings indicate (Fig. 3a–f), mutant MEPM cells (Fig. 3d–f) take longer to close the wound than WT cells (Fig. 3a–c). This effect was quantified by image-analysis measures of confluency (Fig. 3g), from which a 33% reduction in the average speed of wound-fronts, from 6 to 4 µm/h, could be established (Fig. 3i). We used PIV analysis to quantify overall cell-motility within the entire microscopic field. The analysis indicated that MEPM cells remained motile even after closure of the wound (Fig. 3h), and the overall motile activity of *Specc1l*^*cGT/ΔC510*^ mutant MEPM cells was reduced by 20%, from 4.4 to 3.5 µm/h (Fig. 3i).

**Figure 4:**
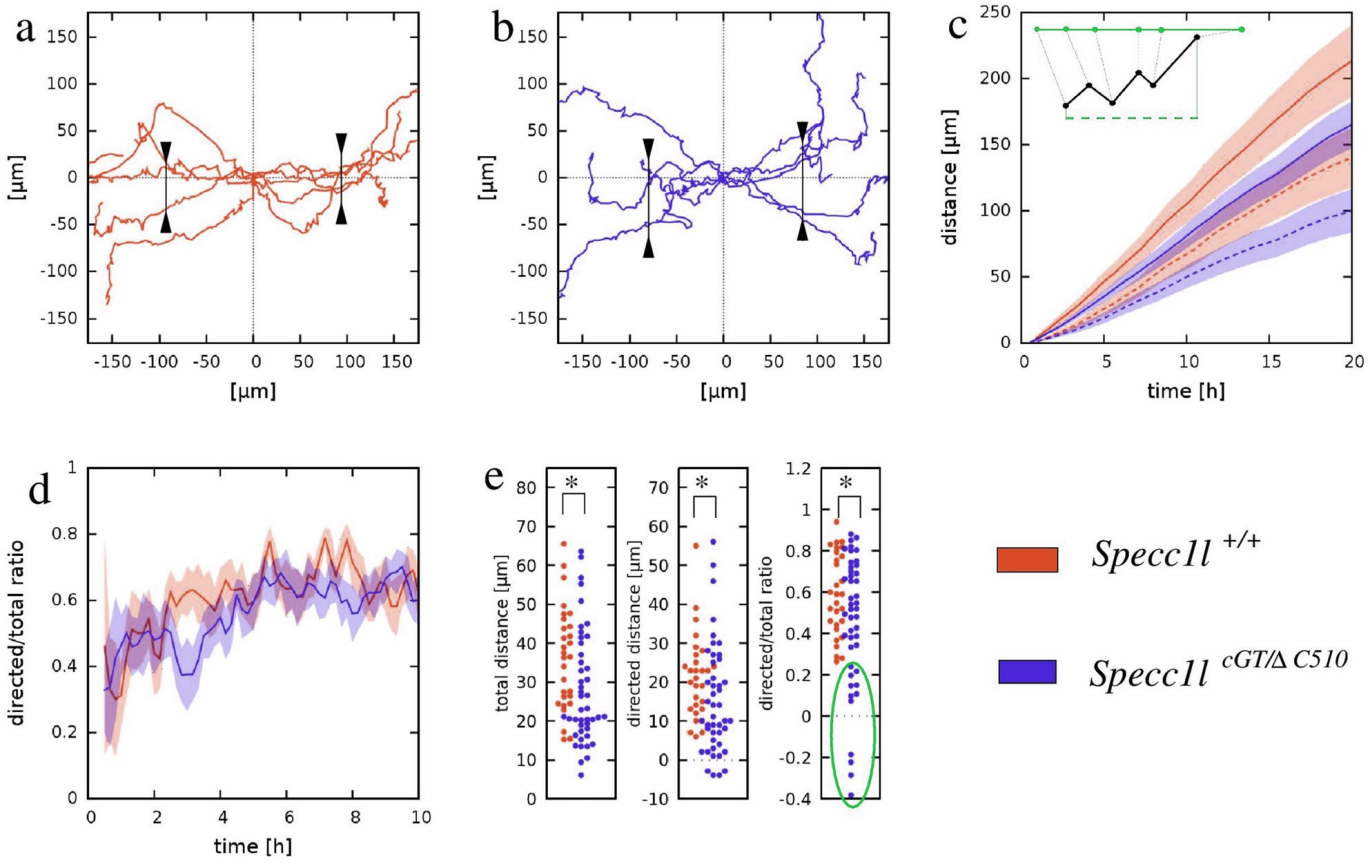
*Specc1l*^*cGT/ΔC510*^ MEPM cells exhibit defects in directional migration. (**a**,**b**) Representative paths of individual cells moving near the wound edge in WT (**a**) and mutant (**b**) cultures. Trajectories were shifted so that each originate at the origin. The WT cells exhibited a more directed movement to the open area compared to the mutant cells, as evidenced by the clustering of trajectories along the horizontal axis. Markers indicate the spread of trajectories: 90% of the trajectories pass through the drawn segments. (**c**) Individual cell trajectories were statistically characterized by their mean total path lengths (solid lines) and net displacements directed into the wound area (dashed lines). The inset demonstrates these concepts with an example trajectory (black). Both measures were plotted as a function of time elapsed, and show a reduction of motile activity in *Specc1l*-mutant MEPM cells. Lines represent an average of 40 cell trajectories, shaded areas indicate SEM. (**d**) The average ratio of the directed (net) displacement and total path length of the 40 cell trajectories, as a function of time. The typically lower values obtained for mutant MEPM cells indicate a guidance defect. (**e**) Statistical analysis of individual cell trajectories using the measures of total displacement, directed displacement and their ratio. The bee swarm plots show these quantities for each tracked cell, evaluated at 10 h culture time. The unguided population delineated by green oval was present only in cultures of *Specc1l-*mutant MEPM cells. The significance of differences was established by pairwise Welsh’s t-tests, yielding p-values of 0.01, 0.05 and 0.03 for the total path length, directed displacement and ratio data sets, respectively.

To identify motility defects at the level of individual cells, 40 WT and 40 *Specc1l*^*cGT/ΔC510*^ mutant MEPM cells were tracked manually as they moved into the wound. Cell trajectories of *Specc1l*^*cGT/ΔC510*^ mutant MEPM cells (Fig. 4a,b) indicated a larger deviation perpendicular to the direction of wound-closure, hence a less-efficient guidance into the wound. Each trajectory was characterized at various timepoints during wound-closure by: (1) the length of total distance migrated (Fig. 4c, solid lines), (2) the net displacement into the wound (Fig. 4c, dashed lines), and (3) the ratio of these two distances as a measure of guidance efficiency (Fig. 4d). While these quantities did not indicate a sudden change in cell behavior, the guidance efficiency of WT MEPM cells improved by ~ 50% during the first 10 h of the wound-closure process (Fig. 4d). Compared to these WT measures, the *Specc1l*^*cGT/ΔC510*^ mutant MEPM cells exhibited a 25% reduction in total distance migrated, and a larger 30% reduction in the net displacements into the wound (Fig. 4c). The larger reduction in directed motility is associated with consistently poor guidance efficiency of mutant MEPM cells (Fig. 4d). Distribution of the three motility measures within the tracked-cell population is quite broad (Fig. 4e). The most conspicuous difference between WT and *Specc1l*^*cGT/ΔC510*^ mutant cultures is the presence of an unguided cell population within the latter (Fig. 4e, green circle). Thus, population-level measures as well as analysis of individual trajectories indicate a substantial role of SPECC1L in both promoting MEPM migration and responding to guidance cues.

### MEPM cells display attributes of collective movement

**Figure 5:**
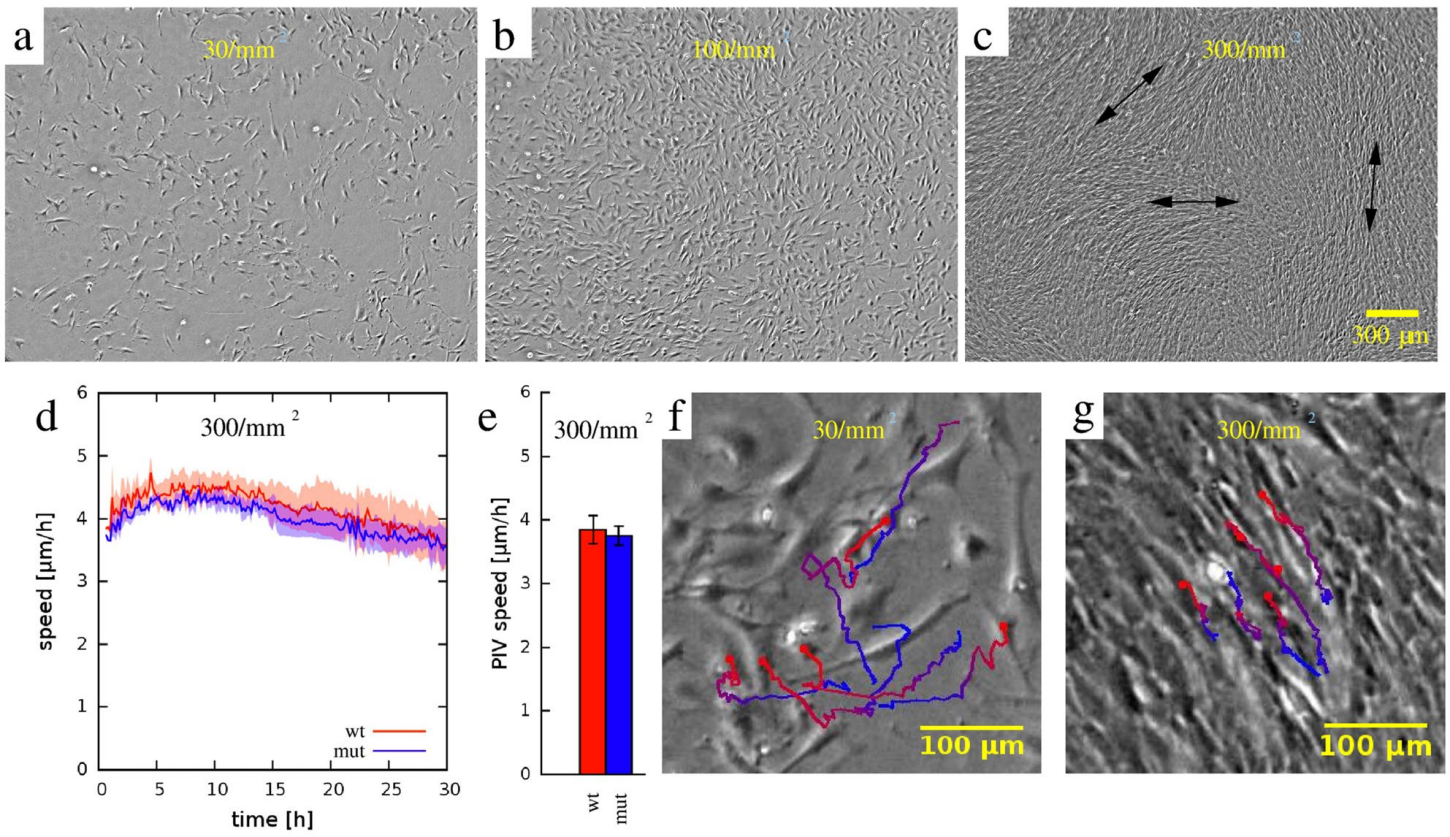
Primary MEPM cells show directional alignment at high density in 2D random motility assays. (**a**–**c**) Representative phase contrast micrographs of wildtype (WT) MEPM cells at low (30/mm^2^), medium (100/mm^2^), and high (300/mm^2^) plating densities. When cultured at high cell densities, MEPM cells exhibited a local directional alignment of cells (arrows). (**d**) The average cell motility speed of high density MEPM cultures demonstrated sustained cell motility despite the high cell density. Velocities were calculated by PIV analysis and averaged over the entire field of view. Orange and blue colors indicate WT and *Specc1l*-mutant cells, respectively. Solid lines are averages of 4 independent fields, shaded areas indicate SEM. (**e**) The average speed of both WT and mutant cells were calculated for the entire recorded time period. Error bars indicate SEM, the difference is not significant. (**f**,**g**) Tracks of individual cells in low (**f**) and high density (**g**) cultures. The random and uncorrelated motility at low cell densities (**f**) became highly aligned at high densities evidenced by the parallel trajectories (**g**). Trajectories are plotted for a time period of 8 h, with red and blue colors indicating earlier and later segments, respectively.

**Figure 6:**
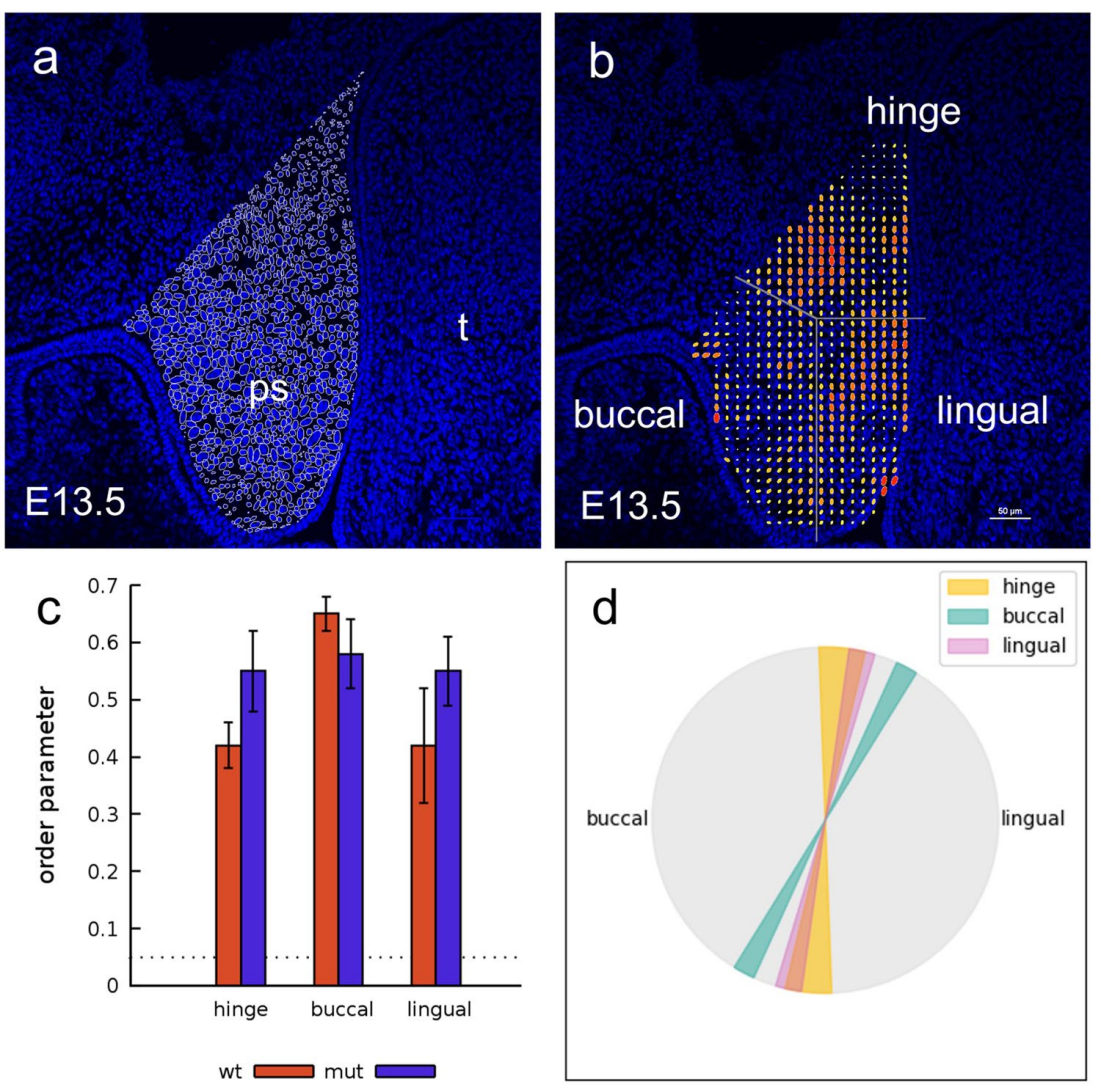
Mesenchymal cell alignment is observed in wildtype and mutant palatal shelves at E13.5. (**a**) Alignment of palate mesenchymal cells is shown by outlining DAPI-labeled nuclei within an epifluorescence micrograph of an E13.5 wildtype embryo palatal shelf (ps). Adjacent nuclei orientation appears in similar directions, and the local prevalent directionality is indicated by yellow lines. t = tongue. (**b**) Adjacent nuclei tend to orient in similar directions, which is quantified by a local order parameter, represented as ellipses. The size of the symbols and warmer colors indicate a greater degree of orientation, while the long axis of the ellipses indicate the prevailing local orientation of nuclei. To statistically characterize the spatial orientation of nuclei across multiple specimens, we distributed the palatal shelves into three regions (hinge, lingual and buccal), as indicated. (**c**) Average alignment of nuclei in the hinge, buccal and lingual regions for wildtype (n = 4) and mutant embryos (n = 4). The dotted line indicates the 99% confidence interval for the null hypothesis assuming independent, uniformly distributed random nuclear orientation. Thus, presented values indicate a statistically significant order (p < 0.001, n = 4), while the differences between regions and between wildtype and mutant embryos is not significant. (**d**) The prevailing orientational order is parallel with the palatal shelf, but it becomes tilted toward the tongue in the buccal region. The difference in orientation is significant (p = 0.005 and p = 0.012 for the buccal vs lingual (p < 0.005) and buccal vs hinge (p < 0.012) regions.

Since our analysis of wound-repair live-imaging studies indicated that *Specc1l*^*cGT/ΔC510*^ mutant cells had defects in speed and guidance, we asked a more fundamental question: do WT MEPM cells display attributes of collective movement? To test this, we recorded WT and *Specc1l*^*cGT/ΔC510*^ mutant MEPM cells in a simple two-dimensional tissue-culture environment (Fig. 5a–c, WT cells; Suppl. Movie 2). We use the cell-front density in the wound of ~ 300 cells/mm^2^ as reference (“high”), and included two lower cell densities of 100 cells/mm^2^ (“medium”) and 30 cells/mm^2^ (“low” or individual cells) for comparison. Interestingly, only when cultured at high density, MEPM cells created ordered and long-range oriented domains reaching up to 1 mm (Fig. 5c). While we could not perform live-imaging at such resolution in embryos, we looked at E13.5 embryonic tissue sections (Fig. 2a,b) for evidence of alignment of palatal shelf mesenchymal cells. We used nuclear shape and orientation as a proxy marker for orientation of the spindle-shaped mesenchymal cells (Fig. 6a,b), and found that palatal shelf mesenchymal cells tend to be aligned with their neighbors (Fig. 6c), consistent with our findings of stream-formation in cultured MEPM cells. In particular, we found that the prevailing orientation of the nuclei is parallel with the vertical axis of the E13.5 palatal shelf (Fig. 6d). The buccal region, however, exhibits a significant orientation toward the tongue at E13.5, just prior to the elevation of the palatal shelves (Fig. 6d). Thus, cultured MEPMs at high density appear ordered similarly to the in vivo palatal mesenchyme.

Analysis of spontaneous MEPM cell motility by PIV analysis, revealed a sustained motile behavior despite the high cell density (Fig. 5d). The average motile speed was not significantly different between WT and mutant cultures (Fig. 5e, ~ 4 µm/h), in contrast to PIV cell speeds observed in wound-repair experiments (Fig. 3i). Trajectories of individually tracked cells revealed a cell-density-dependent ordering (Suppl. Movie 3): cell trajectories of adjacent cells in low-density cultures appeared uncorrelated (Fig. 5f, WT cells), while those in high-density cultures were largely parallel (Fig. 5g, WT cells).

**Figure 7:**
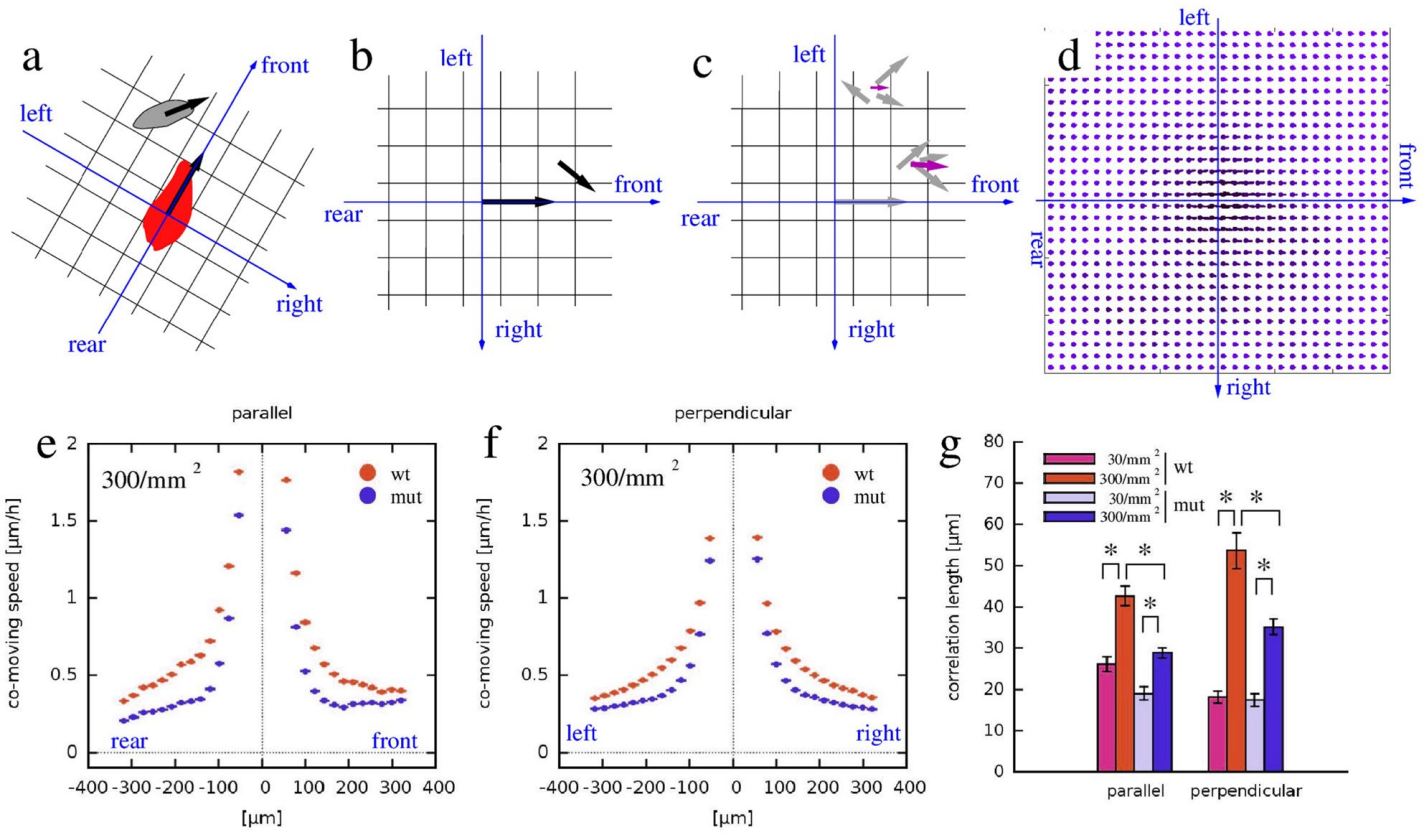
*Specc1l*^*cGT/ΔC510*^ mutant MEPM cells exhibit a collective migration defect in 2D random motility assays. (**a**–**d**) To characterize spatial correlations in cell motility, we determined the average co-moving velocity at various locations relative to an average motile cell. (**a**) For each moving cell (red) we determined the relative position and velocity of every other cell (gray) within the vicinity. (**b**) The velocities were assigned to bins of a coordinate system, co-aligned with the moving cell. (**c**) By repeating this procedure for multiple cells and multiple time points, each bin contained multiple velocity vectors (gray). The average velocity vector in each bin (magenta) was indicative of the spatial correlation: the average of random vectors was close to zero, while it was non-zero when velocities shared a common component. (**d**) The average velocity map thus characterized the typical cell velocities at various locations relative to a moving cell. We sampled this field along the front-rear axis and also along the left–right axis (**e**,**f**). (**e,f**) MEPM cells in high density cultures tended to move locally in the same direction, indicated by positive average co-moving speed values—both along the front-rear (**e**) and lateral (**f**) axes. Data were averaged from 4 independent fields, both for WT (orange) and *Specc1l*-mutant (blue) cells. The co-movement of *Specc1l*-mutant cells, however, diminished at a closer distance than that of WT cells. (**g**) Average correlation lengths, both parallel with and perpendicular to the direction of cell motion, were established by fitting an exponential function on the profiles shown in (**e**,**f**). High-density cultures (orange and blue) showed a significantly increased correlation length compared to low-density cultures (pink and grey). The increase, however, was approximately twice as large in WT cells (pink vs orange) than in *Specc1l*-mutant cells (grey vs blue). Significance values of the differences were established by Welch t-tests, and are summarized in Supplemental Table S2.

To quantify this apparent collective motion of MEPM cells at high density, we adopted a spatial cross-correlation measure^24,25^, which determined the average co-movement speed of cells at various locations (e.g., front, rear, left, right) relative to a moving cell (Fig. 7a–d). Correlation between cell movements was higher when cells were in close vicinity and gradually tapered off as distance increased beyond 300 µm (Fig. 7e,f). This effect was quantified by fitting the co-movement speed profiles with an exponential function, yielding the correlation length as a fitted parameter. Correlation length values indicated a 33% reduction, only at high density, in both the parallel and perpendicular correlation lengths of *Specc1l*^*cGT/ΔC510*^ mutant MEPM cells when compared with WT MEPM cells. This reduction in correlated movement at high cell-densities (Fig. 7g) suggests an impaired ability of mutant cells to form streams.

Collective movement, especially in mesenchymal cells, requires active cell–cell communication involving cell-adhesion molecules, as well as dynamic reorganization of the cytoskeleton to direct movement in response to signals from neighboring cells^24,29,30^. The more pronounced correlation in movement seen in high-density MEPM cultures is consistent with this contact-dependent mechanism. To further support this contact-dependent mechanism, we looked at both expression of adhesion molecules that are involved in collective movement of mesenchymal cells such as the neural crest, as well as reorganization of the actin cytoskeleton during migration. Using a previously published primary MEPM RNAseq dataset^31^, we were able to identify robust expression of all major adhesion molecules identified to be involved in collective migration of mesenchymal cells^32^. These molecules include N-cadherin, RhoA, Rac1, Cdc42, as well as components of Robo/Slit and Eph/EphR signaling (Suppl. Table S1). We also confirmed the presence of cadherins in primary MEPM cells at the protein level, using a pan-Cadherin antibody (Suppl. Fig. S1). Similar to other cell types, primary MEPM cells during migration also organized their actin filaments perpendicular to the direction of migration (Suppl. Fig. S2). Consistent with our previous U2OS data^14^, fewer mutant cells reorganized their actin filaments adequately (Suppl. Fig. S2). Thus, our data indicate that cultured MEPM cells have an innate ability to move collectively.

### Upregulation of PI3K-AKT signaling rescues Specc1l-deficient migration defects

**Figure 8:**
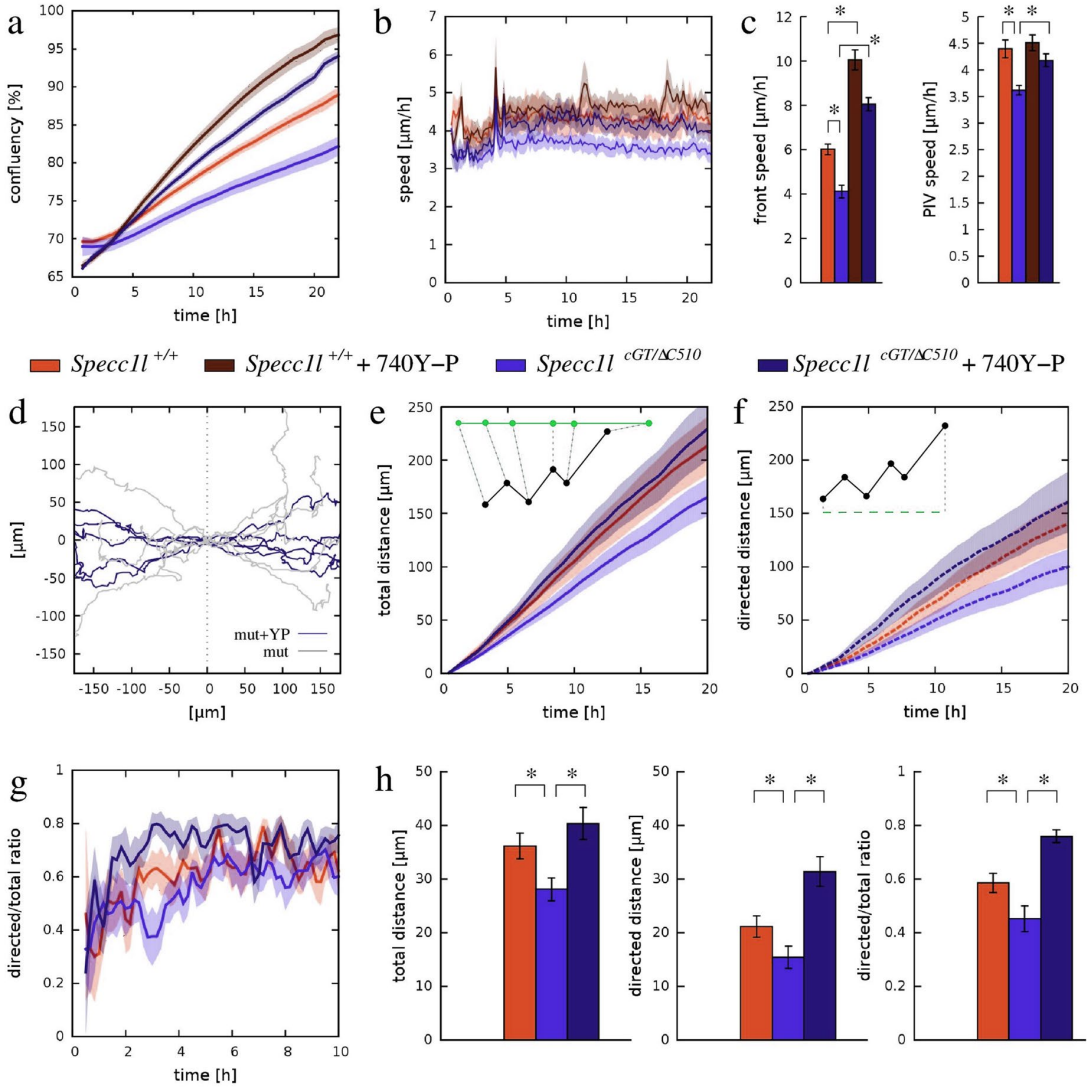
Upregulation of PI3K-AKT signaling rescues both speed and directionality of *Specc1l*^*cGT/ΔC510*^ mutant cells in wound-closure assays. (**a**) Confluency analysis of wound-closure recordings indicated a substantially increased speed of the process as a response of 100 µg/mL 740Y-P, a PI3K activator. Orange and blue colors indicate data obtained from wild-type (WT) and *Specc1l-*mutant cultures, while lighter and darker colors indicate vehicle-treated and 740Y-P treated cultures, respectively. Solid lines represent the average of 13 independent microscopic fields, recorded in 4 independent experiments. Shaded areas indicate SEM. (**b**) Average cell motility speed, calculated by PIV analysis of the wound-closure recordings analyzed in (**a**). The activator 740Y-P induced an increased motile activity, persistent for at least a day, in *Specc1l*-mutant cells. (**c**) Statistical analysis of data presented in (**a**,**b**) indicated that the motility defect of mutant MEPM cells was rescued by the PI3K-AKT activator 740Y-P. Left: average speed of front propagation, calculated within the 1–15 h time interval. The speed-up in wound-closure is significant (p < 5 × 10^−7^ for WT and p < 3 × 10^−10^ for mutant, Welch’s t-test). Right: average speed of cell movements within the entire recorded time period of 24 h. The increase of speed was significant in *Specc1l*-mutant cells (p < 4 × 10^−8^, Welch’s t-test). Error bars represent SEM. (**d**) Trajectories of individual, 740Y-P treated *Specc1l*-mutant MEPM cells (dark blue) was compared with that of untreated MEPM cells (gray). The PI3K-AKT activator enhanced the guidance of cell movements into the wound area. (**e**–**h**) Statistical analysis of individual cell trajectories. Total path length (**e**), net directed displacement into the wound (**f**) and their ratio (**g**) indicated a sustained improvement in the performance of *Specc1l*-mutant MEPM cells. (**h**) Bar charts indicating the cell trajectory measures evaluated at 10 h after the onset of migration. The significance of rescue was established by pairwise Welsh’s t-tests, yielding p-values of 2 × 10^−3^, 2 × 10^−5^, and 4 × 10^−7^ for the total path length, net directed displacement and ratio data sets, respectively. The significance values for the WT-mutant comparison were reported in Fig. 2.

We previously reported that loss of SPECC1L results in decreased PI3K-AKT signaling along with defects in cell-adhesion and cell-shape^18^. Pharmacological activation of the pathway was sufficient to rescue these phenotypes^18^. To test if PI3K-AKT pathway activation could also rescue wound-closure defects in mutant MEPM cells, we repeated the wound-closure experiments reported in Fig. 2 in the presence and absence of 740Y-P (100 µg/mL), a peptide activator of PI3K (Fig. 8). We found that activation of PI3K-AKT pathway did indeed rescue the wound-closure defect in *Specc1l*^*cGT/ΔC510*^ mutant MEPM cultures (Fig. 8a). Specifically, 740Y-P treatment increased the rate of wound-closure in both WT and *Specc1l*^*cGT/ΔC510*^ mutant MEPM cultures by almost twofold (Fig. 8c), even beyond the rate observed in untreated WT MEPM cultures. Interestingly, PIV analysis of 740Y-P treatment did not show an increase in overall WT cell motility, and only a 20% increase in *Specc1l*^*cGT/ΔC510*^ mutant cell motility (Fig. 8b,c). Therefore, we hypothesized that improved cell-guidance into the wound contributed to the improved wound-closure in mutant cultures treated with 740Y-P.

To determine if cell-guidance was improved in the rescue experiments, we compared the trajectories of individual MEPM cells from treated and untreated cultures (Fig. 8d). The dispersion of *Specc1l*^*cGT/ΔC510*^ mutant cells perpendicular to the direction of wound-closure was visibly reduced by PI3K activation, indicating improved guidance into the wound. Statistical evaluation of individual cell-trajectories (Fig. 8e–h) revealed a 40% increase in the total distance migrated (Fig. 8e,h), a twofold increase in the net displacement directed into the wound (Fig. 8f,h), and a 66% increase in guidance efficiency (Fig. 8g,h) in 740Y-P treated *Specc1l*^*cGT/ΔC510*^ mutant MEPM cells. Taken together, the data indicate that PI3K activation can rescue SPECC1L deficiency-related motility defects by improving both cell motility speed and guidance efficiency.

### SPECC1L-dependent collective migration in other cell types

SPECC1L deficiency has been previously shown to result in defects in cell migration in cultured U2OS cells^14,18^. To support a primary role of SPECC1L in collective cell migration, we analyzed time-lapse imaging of control and *SPECC1L*-kd U2OS cells in wound-repair assays, similarly to the MEPM data. We were able to show again in U2OS cells that SPECC1L deficiency resulted in slower wound-closure coupled with poor stream-formation and directional movement (Suppl. Fig. S3). We also confirmed that activation of the PI3K-AKT pathway rescued the cell speed and guidance defects in *SPECC1L*-kd U2OS cells (Suppl. Fig. S4). Interestingly, in contrast to WT MEPM cells, control U2OS cells did not exhibit a significant effect following PI3K-AKT pathway activation. This lack of a marked response likely reflects that U2OS is a cancer cell line with an intrinsically higher than normal level of PI3K-AKT signaling^33–35^. Taken together, these findings point to a common role of SPECC1L in the regulation of cell motility and guidance.

## Discussion

Studies of palatogenesis have demonstrated that remodeling from vertical to horizontal direction is a primary feature of the elevation process^3,6,36^. This remodeling is likely initiated by the medial edge epithelium of the palatal shelf, but driven by the palate mesenchyme. While primary MEPM cells have been used for testing the effect of various inhibitory compounds, growth factors, and gene-expression changes^31,37–42^, only a few studies have investigated the migration of MEPM cells^39,43,44^, and none, to our knowledge, have examined if they can migrate collectively. We identify the presence of stream formation in MEPM cells, a behavior generally considered to be a feature of collective movement^24,25,45^.

We also show that SPECC1L deficiency impaired the ability of palatal mesenchyme cells to move collectively—a result that we posit partially explains the observed delayed and abnormal palate elevation in *Specc1l*^*cGT/ΔC510*^ mutants (Fig. 1)^19^. The defect in collective migration was also validated in U2OS cells, an independent cell line, which strongly supports a primary role for SPECC1L in collective movement.

We previously showed that SPECC1L deficiency leads to reduced PI3K-AKT signaling, and that PI3K-AKT activation was able to rescue cell-shape and cell-adhesion phenotypes in *SPECC1L*-kd U2OS cells^18^. Our analyses now show that activation of PI3K-AKT signaling rescued both cell speed and directionality defects during wound-closure of *Specc1l*^*cGT/ΔC510*^ mutant MEPM cells, as well as in *SPECC1L*-kd U2OS cells. We have shown previously that SPECC1L deficiency leads to a reduction in total AKT levels, rather than just activated phospho-AKT^18^. This reduction in total AKT is at the protein level as RNA levels do not change^18^. Several processes have been identified that play a role in AKT stability, including ubiquitin proteasome-dependent pathway, caspase-mediated cleavage, and caspase-dependent ubiquitination^46^. AKT is known to require translocation to the cell membrane for full activation^47–49^. Thus, it is possible that SPECC1L deficiency affects one or more of these processes involved in AKT stability, resulting in increased AKT degradation. Taken together, our results are consistent with a pro-migratory role for AKT^50^ and suggest that SPECC1L regulates cell migration through PI3K-AKT signaling.

This study represents the first depiction of collective movement characteristics in cultured MEPM cells, and supports the hypothesis that palate elevation involves collective movement of the mesenchyme. In addition to our *Specc1l* mutants, there remain several mouse mutants with elevation defects for which the underlying pathogenic mechanisms have not been fully elucidated^1,8,9,51^. Future analysis of these mutants may benefit from using primary MEPM cell migration as a proxy for mesenchymal remodeling during palatal shelf elevation.

## Supplementary Information

Supplementary Information.

Supplementary Video 1.

Supplementary Video 2.

Supplementary Video 3.

## Data Availability

The live-imaging data that support the findings of this study are available from the corresponding authors upon reasonable request.
