# Supplementary material for: SPECC1L-deficient primary mouse embryonic palatal mesenchyme cells show speed and directionality defects
Source: Sci Rep. 2021 Jan 14;11:1452. doi: 10.1038/s41598-021-81123-9 (PMC7809270; doi:10.1038/s41598-021-81123-9)
Supplement: Supplementary file 1 — Supplementary Information. [file 41598_2021_81123_MOESM1_ESM.pdf]

SPECC1L-deficient primary mouse embryonic palatal mesenchyme cells show speed and directionality defects

Jeremy P. Goering[1§], Dona G. Isai[1§], Everett G. Hall[1§¶], Nathan R. Wilson[1‡], Edina Kosa[1], Luke W. Wenger[1], Zaid Umar[1], Abdul Yousaf[1], Andras Czirok[1*], Irfan Saadi[1*].

[1]Department of Anatomy and Cell Biology, University of Kansas Medical Center, Kansas City, KS.

[§]These authors contributed equally

¶Current address: Clinical Research Training Center, Institute of Clinical and Translational Sciences, Washington University, St. Louis, MO.

‡Current address: Center for Regenerative Medicine, Massachusetts General Hospital, Harvard Medical School, Boston, MA.

*To whom correspondence should be addressed:

Irfan Saadi, PhD

Associate Professor
University of Kansas Medical Center
Department of Anatomy and Cell Biology
3901 Rainbow Blvd., Kansas City, KS 66160
Tel: 913-588-7667
Fax: 913-588-5677
Email: isaadi@kumc.edu

Andras Czirok, PhD

Associate Professor
University of Kansas Medical Center
Department of Anatomy and Cell Biology
3901 Rainbow Blvd., Kansas City, KS 66160
Email: aczirok@kumc.edu

Supplemental Table 1: MEPM expression of genes encoding proteins involved in collective migration of mesenchymal cells

Protein	Gene**	gene_refseq	FPKM* (Unstimulated)
N-Cadherin	***Cdh2***	**NM_007664**	**104.7**
RHOA	***Rhoa***	**NM_016802**	**164.9**
RAC1	***Rac1***	**NM_009007**	**191.8**
CDC42	***Cdc42***	**NM_001243769,NM_009861**	**275.3**
p21 activated kinases (PAKs)	*Pak1*	NM_011035	9.0
	Pak2	**NM_177326**	**34.6**
	Pak3	NM_001195046,NM_001195047,NM_001195048, NM_001195049,NM_008778	10.2
β-PIX	***Arhgef7***	**NM_001113517,NM_001113518,NM_017402**	**16.9**
N-CAM	***Ncam1***	**NM_001081445,NM_001113204,NM_010875**	**36.4**
Nectins	*Nectin1*	NM_021424	6.7
	Nectin2	**NM_001159724,NM_008990**	**46.5**
	Nectin3	**NM_021495,NM_021496,NM_021497**	**22.3**
	Nectin4	NM_001122680,NM_027893	0.2
Afadin	***Afdn***	**NM_010806**	**16.5**
Ponsin	***Sorbs1***	**NM_001034962,NM_001034963,NM_001034964, NM_009166,NM_178362**	**13.5**
Vinculin	***Vcl***	**NM_009502**	**223.7**
Robo ligands	***Robo1***	**NM_019413**	**11.6**
	Robo2	NM_175549	4.4
	Robo4	NM_028783	0.0
Slit receptors	*Slit1*	NM_015748	0.1
	Slit2	**NM_178804**	**100.0**
	Slit3	**NM_011412**	**73.2**
Ephrin A ligands	*Efna1*	NM_001162425,NM_010107	6.7
	Efna2	NM_007909	3.2
	Efna3	NM_007911	0.7
	Efna4	**NM_007910**	**16.1**
	Efna5	**NM_010109,NM_207654**	**14.9**
Ephrin B ligands	***Efnb1***	**NM_010110**	**29.9**
	Efnb2	**NM_010111**	**43.4**
	Efnb3	NM_007911	0.7
Ephrin A receptors	***Epha1***	**NM_023580**	**13.4**
	Epha2	**NM_010139**	**45.8**
	Epha3	**NM_010140**	**14.3**
	Epha4	**NM_007936**	**20.6**
	Epha5	NM_007937	0.1
	Epha6	NM_007938	0.0
	Epha7	**NM_001122889,NM_010141**	**15.9**
	Epha8	NM_007939	0.0
	Epha10	NM_001256432,NM_177671	0.0
Ephrin B receptors	*Ephb1*	NM_001168296,NM_173447	0.1
	Ephb2	**NM_010142**	**19.3**
	Ephb3	**NM_010143**	**27.0**
	Ephb4	**NM_001159571,NM_010144**	**37.4**
	Ephb6	**NM_001146351,NM_007680**	**12.9**

* from Vasudevan *et al.* (2015) supplemental RNAseq data file "elife07186s001.xlsx"

** Genes in bold show high expression

Supplementary Table 2: Significance values, established by Welch t-tests, of the differences among datasets shown in Figure 5g.

**Parallel**				
	wt low density	wt high density	mut low density	mut high density
wt low density	0.500000	0.005978	0.028468	0.163239
wt high density	0.005978	0.500000	0.001777	0.006985
mut low density	0.028468	0.001777	0.500000	0.007666
mut high density	0.163239	0.006985	0.007666	0.500000

**Perpendicular**				
	wt low density	wt high density	mut low density	mut high density
wt low density	0.500000	0.002246	0.386518	0.002792
wt high density	0.002246	0.500000	0.002170	0.014818
mut low density	0.386518	0.002170	0.500000	0.002689
mut high density	0.002792	0.014818	0.002689	0.500000

Figure S1:

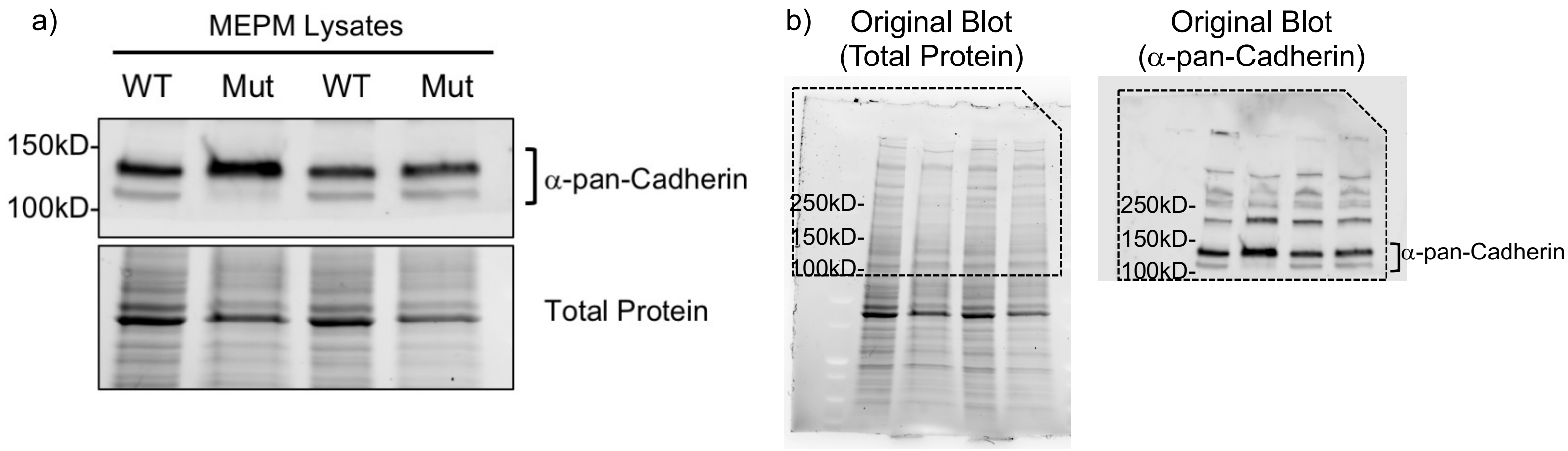

Figure S1: Primary MEPM cells express cadherins. a) Wildtype (WT) and *Specc1l*$^{cGT/\Delta C510}$ mutant (Mut) mouse embryonic palatal mesenchyme (MEPM) cell lysates were used for immunoblotting with anti-pan-Cadherin antibody **(a)** (1:3000; Cell Signaling Technologies, 4068). **b)** Lysates were electrophoresed in 4-15% gradient Mini-Protean TGX Stain-Free precast gels (Bio-RAD, #4568084). After electrophoresis, the gels were exposed to UV light for 45 seconds to develop the total protein signal and imaged **(b, left)** on a ChemiDoc System (Bio-RAD, Hercules, CA) before being transferred onto Immobilon PVDF membranes (EMD Millipore, IPVH00010). The top left of the PVDF membrane **(b, dotted line)** was cut out and blocked in Odyssey Blocking Buffer (Li-Cor, 927-5000) at room temperature for 1 hour. Primary antibody was exposed overnight at 4C. The secondary antibody used was HRP-linked goat anti-rabbit IgG (1:10,000; Cell Signaling Technologies). Femto SuperSignal West ECL reagent (Thermo Scientific, 34095) was used to develop the signal. The original blot for pan-Cadherin is shown **(b, right)**. Data shown are from two WT and Mut littermate pairs.

Figure S2:

Primary MEPM cells

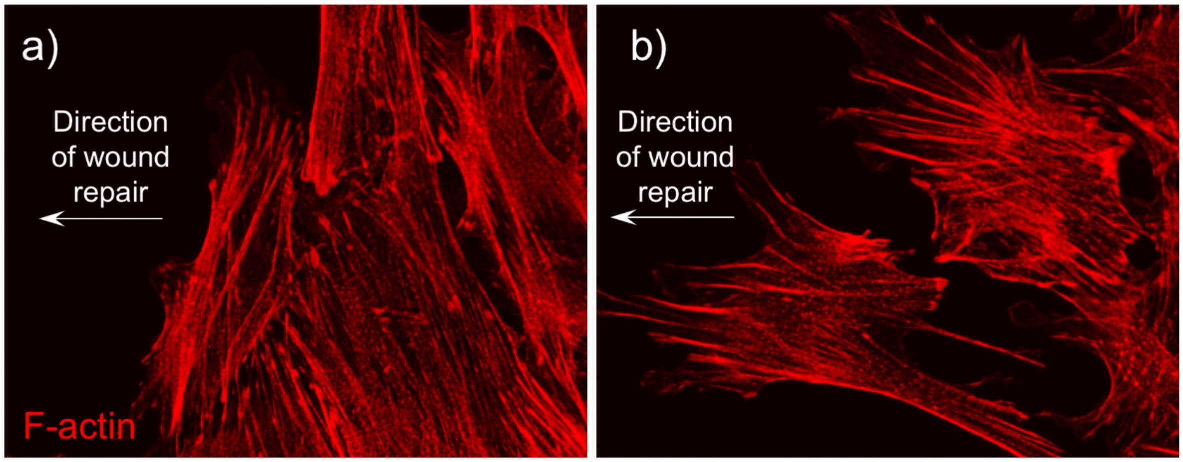

Category 1: Perpendicular* **Category 2: Not Perpendicular**

*Actin filaments aligned perpendicularly to the direction of cell migration into the wound.

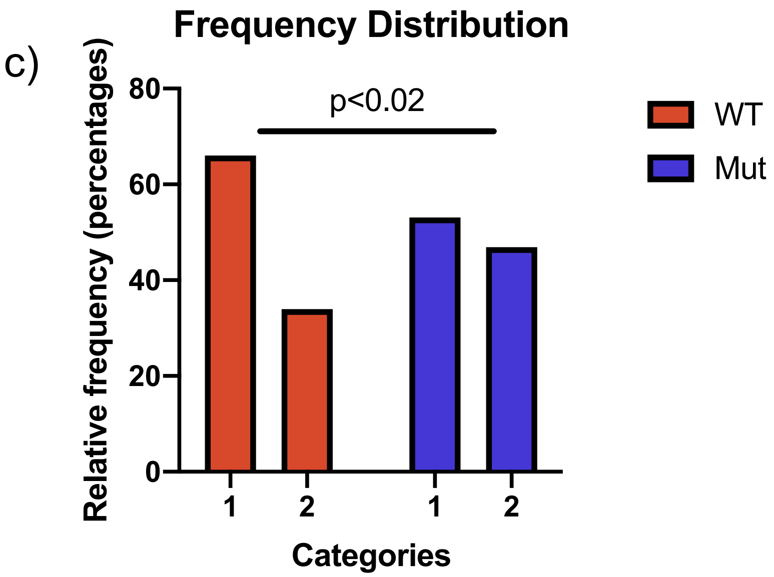

Figure S2: Actin filaments organize perpendicularly to direction of wound repair in migrating primary MEPM cells. a,b) Wildtype (WT) and *Specc1l*$^{cGT/\Delta C510}$ mutant (Mut) MEPM cells were fixed 18 hours after beginning a wound-repair experiment, and stained for filamentous actin (F-actin) using phalloidin. Cells at the migrating cell-front were assessed for actin filament organization, as either perpendicular to the direction of migration (Category 1) or not (Category 2). **c)** Frequency distribution in percentage of WT (n=165) and mutant (n=145) cells assessed in category 1 or 2. There was a statistically significant difference in distribution of mutant cells ($p<0.02$), with an increase in the number of cells with poorly organized actin cytoskeleton (Category 2).

Figure S3:

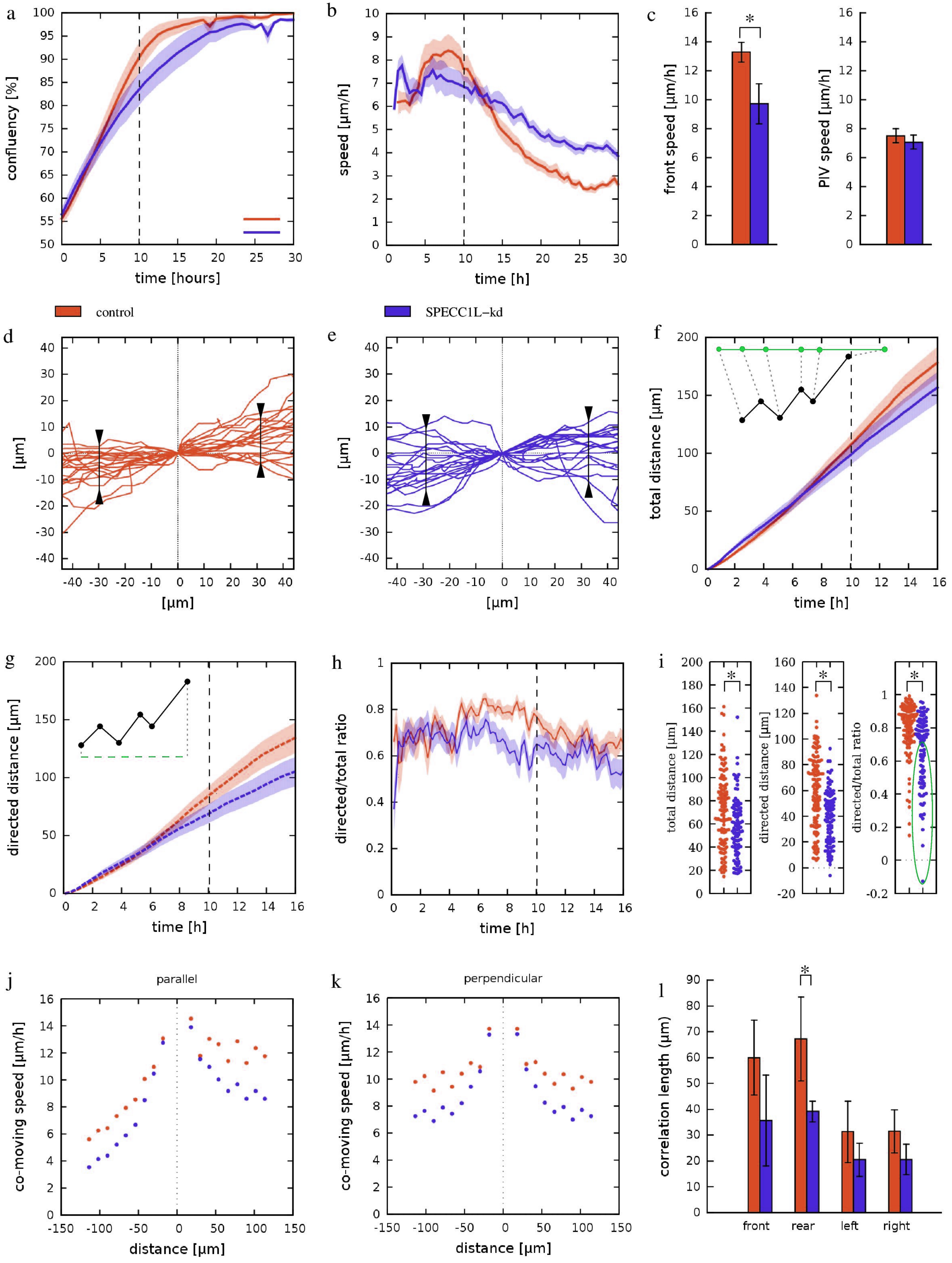

a
confluency [%]
time [hours]
b
speed [µm/h]
time [h]
c
front speed [µm/h]
PIV speed [µm/h]
*
control
SPECC1L-kd
d
e
[µm]
f
total distance [µm]
time [h]
g
directed distance [µm]
time [h]
h
directed/total ratio
time [h]
i
total distance [µm]
directed distance [µm]
directed/total ratio
j
parallel
co-moving speed [µm/h]
distance [µm]
k
perpendicular
co-moving speed [µm/h]
distance [µm]
l
correlation length (µm)
front
rear
left
right

Figure S3: *SPECC1L*-kd U2OS cells show defects in directional migration. a) Confluence analysis of wound-repair experiments indicated a delayed wound closure in *SPECC1L*-kd cell cultures (blue) when compared to control cells (orange). By 10h (dashed vertical line) control U2OS cells closed more than 75% of the wound. Solid lines represent the average of 17 independent microscopic fields, recorded in 4 distinct sets of experiments. Shaded areas indicate SEM. **b)** Average cell motility speed, calculated by PIV analysis of the 17 wound-closure recordings analyzed in panel (a). The motility of both control and *SPECC1L*-kd cells dropped as the wound closed. **c)** Statistical analysis of data presented in panels (a-b) indicated that while the wound-closure of *SPECC1L*-kd cells was significantly impaired in comparison to control U2OS cells ($p<5.5x10^{-6}$, Welch's t-test, left), there is no such profound difference in motile speed (right). Both the average speed of the wound-front and the average speed of cell movements were calculated within the 1h-15h time interval, prior to wound closure. Error bars represent SEM. **d-e)** Representative paths of individual cells moving near the wound-edge in control (d) and SPECC1L-kd (e) cultures. As in Fig. 2, trajectory heads were shifted to the origin, and 90% of the trajectories passed through the segments indicating the lateral spread of trajectories. Control cells exhibit a more directed movement to the open area compared to *SPECC1L*-kd cells. **f-h)** Statistical analysis of individual cell trajectories, in terms of total path-length (f), net directed displacement into the wound (g) and their ratio (h). Measures were plotted as a function of time elapsed, and indicate a reduction of both motile activity and directional guidance in *SPECC1L*-kd U2OS cells. Lines represent averages of 138 and 126 cells from control and *SPECC1L*-kd cultures, respectively. Shaded areas indicate SEM. Vertical dashed lines indicate the 10h timepoint, when the wound was almost closed. **i)** Statistical analysis of individual cell trajectories using the measures of total distance, directed displacement and their ratio, evaluated at 10h culture timepoint. The unguided population, delineated by blue ellipse, is more numerous in *SPECC1L*-kd cultures. The significance of differences was established by pairwise Welsh's t-tests, yielding p-values of $1.2x10^{-4}$, $2x10^{-9}$ and $1.1x10^{-7}$ for the total path length, directed displacement and ratio data sets, respectively. **j-k)** Collective movement of U2OS cells, indicated by average co-moving speed profiles (See Fig. 3) -- both along the front-rear (j) and lateral (k) axes. Data were averaged from 18 independent fields, both for control and *SPECC1L*-kd cells. Synchronized movement between *SPECC1L*-kd cells diminished at a closer distance than that of control cells. **l)** Average correlation lengths, both along directions parallel with and perpendicular to the direction of cell motion, were established by fitting an exponential function on the profiles shown in panels (j, k). Significance of the differences were established by Welch t-tests ($p<0.05$).

Figure S4:

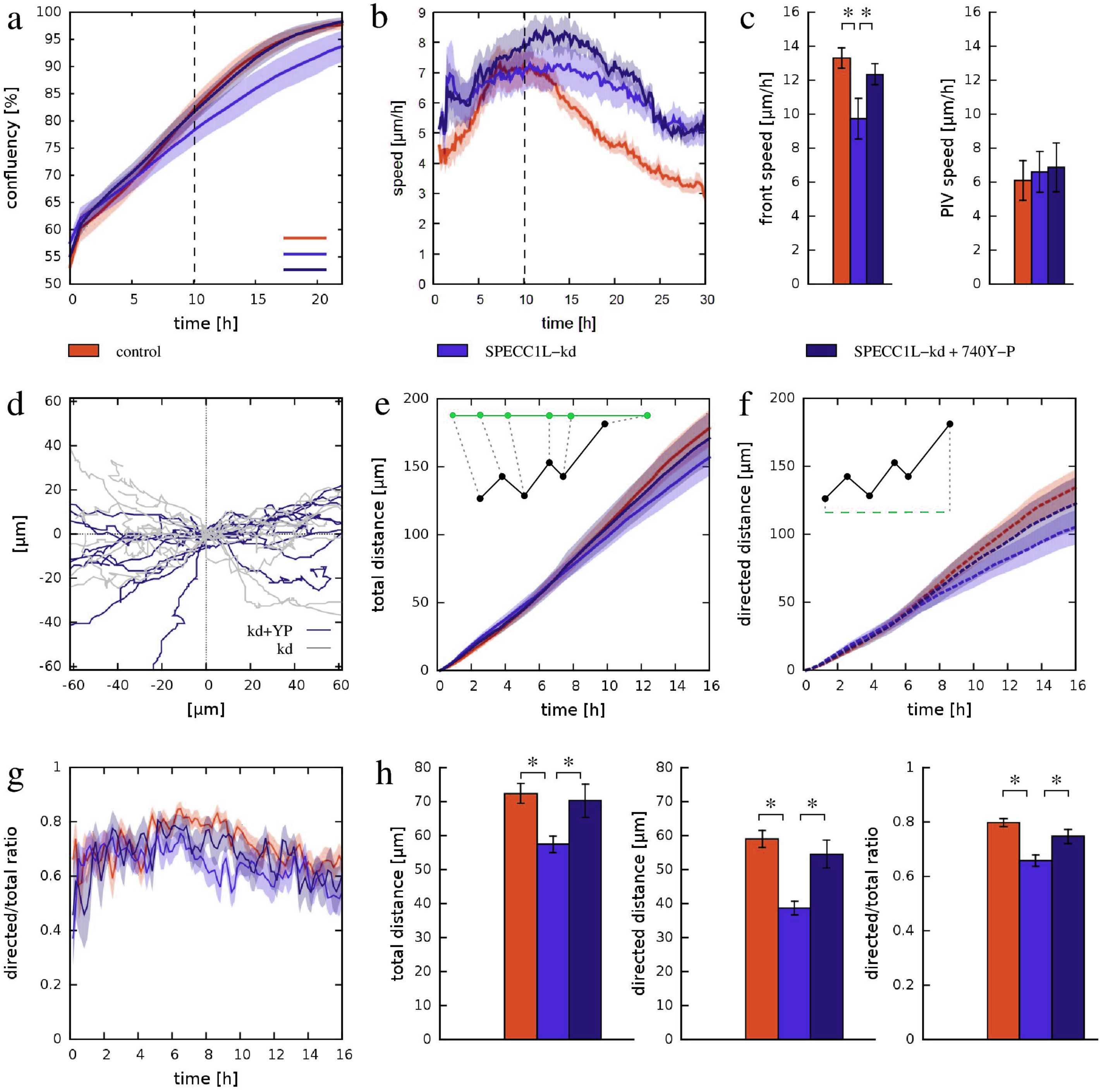

a
confluency [%]
time [h]
b
speed [µm/h]
time [h]
c
front speed [µm/h]
PIV speed [µm/h]
control
SPECC1L–kd
SPECC1L–kd + 740Y–P
d
[µm]
kd+YP
kd
e
total distance [µm]
time [h]
f
directed distance [µm]
time [h]
g
directed/total ratio
time [h]
h
total distance [µm]
directed distance [µm]
directed/total ratio

Figure S4: Upregulation of PI3K-AKT signaling rescues both speed and directionality of *SPECC1L*-kd U2OS cells in wound-closure assays. a) Confluence analysis of wound-repair experiments indicated that 100μg/ml 740Y-P enhances the wound-closure of *SPECC1L*-kd cells (dark blue), compared to untreated *SPECC1L*-kd cultures (blue). The improvement is enough to eliminate the difference with control cells (orange). Solid lines represent the average of 17 independent microscopic fields, recorded in 4 distinct setd of experiments. Shaded areas indicate SEM. **b)** Average cell speed as a function of time, calculated from the 17 microscopic fields analyzed in panel (a). Solid lines represent averages, shaded areas indicate SEM. The PI3K-AKT activator elicited a sustained increase in the speed of *SPECC1L*-kd cells during the wound-closure process. **c)** Statistical analysis of wound-front propagation (left) and cell motility (right) speed data. Wound-front speed was obtained by linear fits over the confluency data within the 1h - 15h time-interval. Cell motility speed was obtained by averaging in the same time window. Error bars represent SEM. Significant differences are indicated by asterisks (control vs *SPECC1L*-kd: $p<5.5x10^{-6}$, 740Y-P treatment: $p<3.6x10^{-5}$). **d)** Representative trajectories of individual cells moving near the wound-edge in 740Y-P-treated *SPECC1L*-kd cultures (dark blue). As a comparison, trajectories of untreated kd cells are plotted in grey. **e-g)** Statistical analysis of individual cell-trajectories, in terms of total path-length (e), net directed displacement into the wound (f) and their ratio (g). The PI3K-AKT activator improved each of these measures for *SPECC1L*-kd cultures (dark blue vs blue). Lines represent averages of 138, 126 and 73 cells from control, untreated *SPECC1L*-kd cultures, and 740Y-P-treated *SPECC1L*-kd cultures, respectively. Shaded areas indicate SEM. **h)** Statistical analysis of individual cell-trajectories using the measures of total path-length, directed displacement and their ratio, evaluated at 10h culture timepoint. The significance of 740Y-P treatment was established by pairwise Welsh's t-tests, yielding p-values of 0.02, $6.6x10^{-4}$ and 0.01 for the total path length, directed displacement and ratio data sets, respectively. Error bars indicate SEM.

Supplemental Movie 1. Phase contrast time-lapse recordings of wound-repair assays. Primary MEPM cells were obtained from wildtype (WT, top), *Specc1l*$^{cGT/\Delta C510}$ mutant (MUT, middle) and mutant treated with 100 μg/ml 740 Y-P, an AKT activator (MUT+YP, bottom). The delay in wound closure (middle) can be rescued by administration of the 740 Y-P AKT activator (bottom).

Supplemental Movie 2. Phase contrast time-lapse recordings of primary MEPM cells within a 2D motility arena. Cultures with a higher seeding density (right) develop more robust orientational order than cultures with lower cell density (left). Orientational order is more robust in MEPM cultures obtained from wild-type embryos (top) than in cultures established from *Specc1l*$^{cGT/\Delta C510}$ mutant embryos (bottom).

Supplemental Movie 3. Trajectory analysis of MEPM cells. Trajectories of a few selected MEPM cells in medium (left) and high (right) density cultures. Trajectory segments are progressively colored using a red-to-blue palette, with red indicating the beginning and blue indicating the end.
